# Assessment of the factorial validity and reliability of the ALSFRS-R: a revision of its measurement model

**DOI:** 10.1007/s00415-017-8538-4

**Published:** 2017-06-12

**Authors:** Leonhard A. Bakker, Carin D. Schröder, Michael A. van Es, Paul Westers, Johanna M. A. Visser-Meily, Leonard H. van den Berg

**Affiliations:** 1 Department of Neurology, Brain Centre Rudolf Magnus, University Medical Centre Utrecht, Heidelberglaan 100, 3584 CX Utrecht, The Netherlands; 2 Centre of Excellence for Rehabilitation Medicine, Brain Centre Rudolf Magnus, University Medical Centre Utrecht and De Hoogstraat Rehabilitation, Utrecht, The Netherlands; 3 Department of Rehabilitation, Physical Therapy Science, and Sports Medicine, Brain Centre Rudolf Magnus, University Medical Centre Utrecht, Utrecht, The Netherlands; 4 Julius Center for Health Sciences and Primary Care, University Medical Center Utrecht, Utrecht, The Netherlands

**Keywords:** Amyotrophic lateral sclerosis, Amyotrophic lateral sclerosis functional rating scale-revised, Confirmatory factor analysis

## Abstract

The amyotrophic lateral sclerosis functional rating scale-revised (ALSFRS-R) is a widely used primary outcome measure in amyotrophic lateral sclerosis (ALS) clinical practice and clinical trials. ALSFRS-R items cannot, however, validly be summed to obtain a total score, but constitute domain scores reflecting a profile of disease severity. Currently, there are different measurement models for estimating domain scores. The objective of the present study is, therefore, to derive the measurement model that best fits the data for a valid and uniform estimation of ALSFRS-R domain scores. Data from 1556 patients with ALS were obtained from a population-based register in The Netherlands. A random split of the sample provided a calibration and validation set. Measurement models of the ALSFRS-R were investigated using both exploratory factor analyses and confirmatory factor analyses. The measurement model with a four-factor structure (i.e., bulbar, fine motor, gross motor, and respiratory function), with correlated factors and cross-loading items on dressing and hygiene and turning in bed and adjusting bed clothes on both motor function scales, provided the best fit to the data in both sets. Correlation between factors ranged from weak to modest, confirming that the ALSFRS-R constitutes a profile of four clinically relevant domain scores rather than a total score that expresses disease severity. The internal consistency of the four domain scores was satisfactory. Our revision of the measurement model may allow for a more adequate estimation of disease severity and disease progression in epidemiological studies and clinical trials.

**Electronic supplementary material:**

The online version of this article (doi:10.1007/s00415-017-8538-4) contains supplementary material, which is available to authorized users.

## Introduction

Amyotrophic lateral sclerosis (ALS) is a progressive neurodegenerative disorder of the motor neurons for which there is currently no effective treatment. Disease progression in ALS is characterized by loss of physical function in various domains, i.e., the bulbar, fine and gross motor, and respiratory domain. The amyotrophic lateral sclerosis functional rating scale (ALSFRS) [1, 2] and its revised version (ALSFRS-R) [3] use this loss of function as a marker for disease severity and disease progression. To date, the ALSFRS-R is the most widely applied rating scale in clinical practice and clinical trials as primary or secondary outcome measure. Moreover, it has been translated into various languages [4–8] and adapted for administration to patients via internet [9], administration to patients and caregivers via telephone [10–12], and for self-administration [13].

The ALSFRS-R has demonstrated good criterion-related validity, and the inter-rater, intra-rater, and test–retest reliabilities of the ALSFRS-R are excellent [3, 7, 10, 14]. Recent studies have examined the factorial validity, i.e., the extent to which items measure the intended construct, of the ALSFRS-R using exploratory factor analyses [15], confirmatory factor analyses [15–17] and item response theory analyses [15–17], and have shown that its items do not constitute a total score, a general severity score, but rather a profile of domain scores [15–17]. Hence, the ALSFRS-R domain scores and a consistent strategy to estimate them are of special importance. In the literature, however, there appears to be a divide between those who use a measurement model with a four-factor structure (i.e., bulbar, fine and gross motor, and respiratory) [3, 4, 7, 17, 18], as hypothesized by the developers of the ALSFRS-R, and those who use an alternative measurement model with a three-factor structure, which combines fine and gross motor domains into one motor domain [5, 15, 16]. Consequently, there is currently not one distinct strategy for estimating ALSFRS-R domain scores.

The application of various measurement models of the ALSFRS-R could give rise to inconsistent results in the literature. The primary objective of the present study is, therefore, to assess the factorial validity of the ALSFRS-R in a large sample of patients with ALS, to derive a measurement model that describes the data best for a valid and uniform estimation of ALSFRS-R domain scores. Furthermore, the internal consistency of these domain scores will be assessed.

## Methods

### Sample

ALSFRS-R data of patients who fulfilled the diagnostic criteria for possible, probable laboratory-supported, probable, and definite ALS, according to the revised El Escorial criteria [19], were obtained from the population-based register in the Netherlands for the cohort 2006–2015. This register was approved by the UMC Utrecht medical ethics review committee.

To obtain the broadest possible cross section and avoid dependency in the data, only the most recent observation per individual was included in the study. The sample (*n* = 1556) was split randomly into a calibration set (S1) and a validation set (S2).

### Amyotrophic lateral sclerosis functional rating scale-revised

The ALSFRS-R is a disease-specific 12-item instrument that measures the extent to which patients with ALS are capable of performing functional activities independently [3]. The questionnaire is structured on a 5-point scale ranging from 4 to 0, where 4 indicates no loss of function and 0 total loss of function. The ALSFRS-R was developed to comprise four scales, each measuring one domain affected by the disease.

### Statistical analyses

Exploratory factor analyses (EFA) of ordered categorical data with orthogonal (Varimax) and oblique (Promax) rotations were performed on the raw data of S1.

Confirmatory factor analyses (CFA) were first conducted on the data of S1 and subsequently cross-validated in S2. Given the ordered categorical response format of the ALSFRS-R, CFA should be performed with the weighted least square mean- and variance-adjusted (WLSMV) estimator. However, it is impossible to compare non-nested, i.e., three-factor and four-factor, models using the WLSMV estimator. Using a simulation study, Rhemtulla and colleagues demonstrated that for ordered categorical data with five or more response categories, a robust maximum likelihood estimator, such as the maximum likelihood mean- and variance-adjusted (MLMV) estimator, can be used to obtain acceptable estimates [20], thus facilitating direct comparison of models based on Bayesian Information Criterion (BIC). The different models of the ALSFRS-R were, therefore, examined with both estimators.

Goodness-of-fit was evaluated using the *χ* ^2^ statistic of exact fit, comparative fit index (CFI), Tucker–Lewis index (TLI), and root mean square error of approximation (RMSEA). For acceptable fit, TLI and CFI should be >0.90, and RMSEA <0.08. Non-nested models were compared with BIC. Nested models were compared with a Δ*χ* ^2^ test.

CFA-based estimation of reliability is considered a more adequate method for calculating scale reliability than the traditionally used coefficient alpha [21]. Therefore, scale reliabilities are estimated using parameter estimates of the optimal CFA model.

Data screening and descriptive statistical analyses were conducted in Rstudio [22]. To assess potential bias due to missing data, both complete case and multiple imputation analyses were performed. Multiple imputation, EFA, and CFA were performed in M*plus* Version 7 [23].

### Competing confirmatory factor analytic (CFA) models of the ALSFRS-R

The first set of models (1a–1d) to be evaluated expressed the hypothesis that ALSFRS-R items constitute four domains. The first model (1a) specified a measurement model with uncorrelated factors and the second (1b) a less constrained measurement model with correlated factors. Subsequent models were respecified based on modification indices (MIs), which provide the expected drop in *χ* ^2^ if a parameter is freely estimated, and theoretical knowledge.

The second set of models (2a–2d) expressed the hypothesis that ALSFRS-R items constitute three domains. Again, the first model (2a) specified a measurement model with uncorrelated factors and the second (2b), a measurement model with correlated factors. Subsequent models were also respecified based on MIs and theoretical knowledge.

The specification of cross-loading items was considered acceptable when an item comprised a combination of functions, while the specification of correlated errors was considered acceptable when respective items had similar content.

**Fig. 1:**
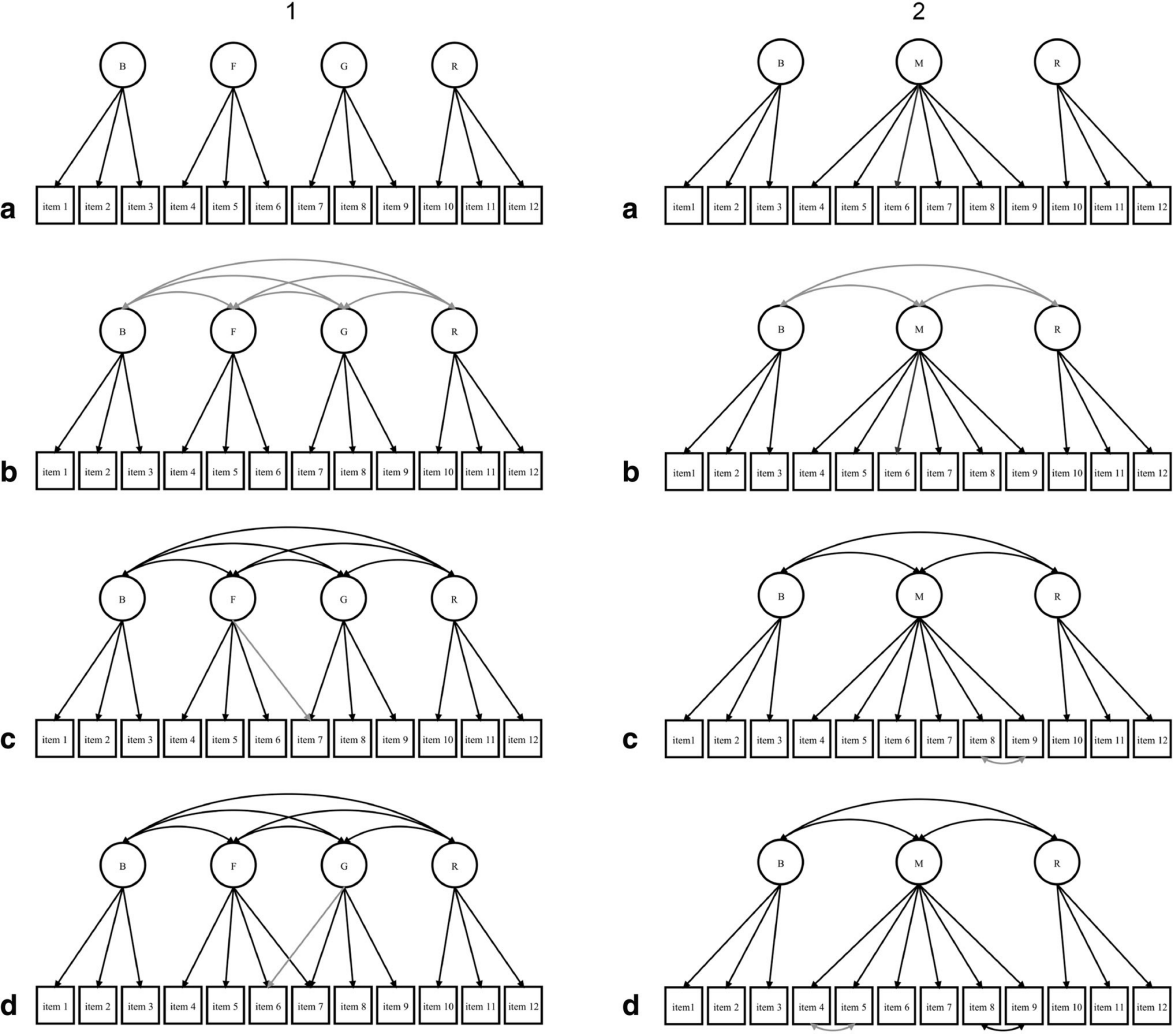
Path models of measurement models of the ALSFRS-R. *Lighter arrows* indicate the parameters that were added to the previous models; *1* speech, *2* salivation, *3* swallowing, *4* handwriting, *5* cutting food and handling utensils, *6* dressing and hygiene, *7* turning in bed and adjusting bed clothes, *8* walking, *9* climbing stairs, *10* dyspnea, *11* orthopnea, *12* respiratory insufficiency, *B* bulbar function, *F* fine motor function, *G* gross motor function, *M* motor function, *R* respiratory function

Path diagrams of competing models are depicted in Fig. 1. M*plus* inputs of both optimal measurement models (1d, 2d) are provided in Online Resources 1 and 2.

## Results

### Sample characteristics

Table 1 presents the demographic and clinical characteristics of both the complete study sample (*N* = 1556) and the two samples that were obtained by a random split of the data (*N*_S1_ = 788, *N*_S2_ = 788). As shown in Table 1, the two samples were comparable.

**Table 1:** Demographic and clinical characteristics of patients

	Sample	S1	S2
*n*	1556	778	778
Sex, *n* (%)			
Female	637 (40.9)	321(41.3)	316 (40.6)
Male	919 (59.1)	457 (58.7)	462 (59.4)
Site of onset, *n* (%)			
Bulbar	511 (32.8)	249 (32.0)	262 (33.7)
Spinal	1039 (66.8)	528 (67.9)	511 (65.7)
Missing	6 (0.4)	1 (0.1)	5 (0.6)
El Escorial diagnosis, *n* (%)			
Definite ALS	288 (18.5)	144 (18.5)	144 (18.5)
Probable ALS	663 (42.6)	316 (40.6)	347 (44.6)
Probable ALS lab supported	277 (17.8)	150 (19.2)	127 (16.3)
Possible ALS	328 (21.1)	168 (21.6)	160 (20.6)
Age, mean (sd)	64.1 (11.0)	64.3 (11.2)	63.9 (10.8)
Age at onset, mean (sd)	62.0 (11.3)	62.3 (11.5)	61.6 (11.0)
ALSFRS-R, mean (sd)^a^	35.1 (8.9)	34.9 (9.0)	35.3 (8.7)

*ALSFRS-R* amyotrophic lateral sclerosis functional rating scale-revised, *S1* calibration set, *S2* validation set

^a^Raw total score

### Exploratory factor analysis (EFA)

To explore the measurement model of the ALSFRS-R EFA for four- and three-factor solutions were performed.

The EFA of the three-factor solution produced a poor model fit. Both orthogonal and oblique rotations of the three-factor solutions yielded uninterpretable patterns of factor loadings (*χ* ^2^ = 550.20, *df* = 33, *p* < 0.001, RMSEA = 0.14).

The EFA of the four-factor solution gave a better model fit (*χ* ^2^ = 73.21, *df* = 24, *p* < 0.001, RMSEA = 0.05). Both orthogonal and oblique rotations revealed a pattern of factor loadings that could be interpreted as representing bulbar, fine motor, gross motor, and respiratory function. However, the item on dressing and hygiene loaded onto two factors with orthogonal rotation. Furthermore, the item on turning in bed and adjusting bed clothes loaded onto two factors with both orthogonal and oblique rotation. Factor loading patterns of both rotations are presented in Table 2.

**Table 2:** Factor loadings of exploratory factor analyses

Indicator	Orthogonal				Oblique			
	V1	V2	V3	V4	P1	P2	P3	P4
Speech	**0.88**	0.12	0.13	0.10	**0.92**	0.03	−0.06	0.02
Salivation	**0.84**	−0.01	0.14	0.02	**0.88**	−0.10	−0.01	−0.03
Swallowing	**0.87**	0.16	0.29	0.09	**0.86**	0.06	0.13	−0.03
Writing	0.02	**0.86**	0.10	0.25	−0.08	**0.95**	0.00	−0.06
Cutting food and handling utensils	0.15	**0.89**	0.11	0.25	0.05	**0.98**	−0.02	−0.07
Dressing and hygiene	0.09	**0.76**	0.12	**0.55**	−0.01	**0.70**	−0.04	0.35
Turning in bed and adjusting bedclothes	0.18	**0.58**	0.16	**0.69**	0.10	**0.41**	−0.01	**0.59**
Walking	0.03	0.28	0.14	**0.92**	−0.05	−0.05	−0.03	**1.02**
Climbing stairs	0.08	0.33	0.20	**0.89**	−0.01	0.02	0.03	**0.95**
Dyspnea	0.15	0.03	**0.81**	0.05	−0.02	−0.06	**0.89**	−0.09
Orthopnea	0.26	0.11	**0.79**	0.17	−0.04	−0.02	**0.81**	0.04
Respiratory insufficiency	0.16	0.28	**0.46**	0.29	0.03	0.18	**0.42**	0.18

Bold type indicates factor loadings >0.40

*P1–P4* promax rotated loadings, *V1–V4* varimax rotated loadings

### Testing competing confirmatory factor analytic (CFA) models of the ALSFRS-R

CFA were performed with WLSMV and MLMV estimators. The two analyses produced a similar pattern of results. Furthermore, potential bias due to missing data was assessed with multiple imputation analyses yielding similar results. These are provided in Online Resource 3.

Table 3 shows fit indices of competing measurement models. Four models were tested in each set of models. In measurement models with a four-factor structure, model fit was poor in the initial model (1a), but improved in subsequent models after the specification of correlated factors and cross-loading items on dressing and hygiene and turning in bed and adjusting bed clothes in the optimal model (1d). In measurement models with a three-factor structure, model fit was poor in the initial model (2a), but improved in subsequent models after the specification of correlated factors and correlated errors between items on walking and climbing stairs and writing and cutting food and handling utensils in the optimal model (2d). For both sets of measurement models, all less constrained models had a significant improvement over more constrained models (*p* < 0.0001).

**Table 3:** Fit statistics for confirmatory factor analyses

Estimator	Model	*χ* ^2^		*df*		CFI		TLI		RMSEA		BIC	
		S1	S2	S1	S2	S1	S2	S1	S2	S1	S2	S1	S2
MLMV	1a	863.90	781.88	54	54	0.76	0.73	0.70	0.73	0.14	0.13	23620	23553
	1b	444.47	400.01	48	48	0.88	0.85	0.84	0.85	0.10	0.10	23022	23040
	1c	253.27	220.37	47	47	0.94	0.95	0.91	0.93	0.08	0.07	22738	22777
	1d	152.95	134.41	46	46	0.97	0.97	0.95	0.96	0.06	0.05	22594	22655
	2a	732.83	701.17	54	54	0.80	0.80	0.75	0.76	0.13	0.13	23435	23436
	2b	610.05	600.23	51	51	0.83	0.83	0.78	0.78	0.12	0.12	23240	23301
	2c	330.32	312.85	50	50	0.90	0.92	0.89	0.89	0.09	0.08	22830	22888
	2d	226.89	230.11	49	49	0.93	0.95	0.93	0.93	0.07	0.07	22680	22771
WLSMV	1a	6312.91	4671.77	54	54	0.73	0.76	0.67	0.71	0.39	0.34		
	1b	733.29	632.27	48	48	0.97	0.97	0.96	0.96	0.14	0.13		
	1c	431.89	345.65	47	47	0.98	0.99	0.98	0.98	0.10	0.09		
	1d	233.00	199.75	46	46	0.99	0.99	0.99	0.99	0.07	0.07		
	2a	1312.38	998.22	54	54	0.95	0.95	0.93	0.94	0.18	0.15		
	2b	922.86	860.71	51	51	0.96	0.96	0.95	0.95	0.15	0.15		
	2c	531.94	458.15	50	50	0.98	0.98	0.97	0.97	0.11	0.10		
	2d	341.07	325.90	49	49	0.99	0.99	0.98	0.98	0.09	0.09		

*BIC* Bayesian information criterion, *CFI* comparative fit index, *MLMV* maximum likelihood means and variance, *RMSEA* root mean square error of approximation, *S1* calibration set, *S2* validation set, *TLI* Tucker–Lewis index, *WLSMV* weighted least squares means and variance

A comparison of BIC values in Table 3 shows that the four-factor model with cross-loading items (1d) has a lower BIC value than the three-factor model with correlated errors (2d), indicating that the former model has a better fit to the data. Models tested in S1 were cross-validated in S2. Furthermore, Table 3 demonstrates that patterns in S2 were similar to patterns in S1.

Table 4 shows fully standardized factor loadings from model 1d in S2. Inspection of the estimates reveals that there is quite some variation between factor loadings, indicating that certain items contribute more to their respective domain score than others. Furthermore, correlations between factors range from weak to modest, indicating that ALSFRS-R subscales do not constitute one overall severity score.

**Table 4:** Estimates of the optimal confirmatory factor analytic model

Domains, items	Reliability	95% CI	Factor loadings, correlations	95% CI
Bulbar function	0.87	0.85–0.89		
Speech			0.89	0.87–0.92
Salivation			0.83	0.79–0.86
Swallowing			0.94	0.92–0.96
Fine motor function	0.90	0.89–0.92		
Writing			0.87	0.85–0.90
Cutting food and handling utensils			0.95	0.92–0.97
Dressing and hygiene			0.68	0.65–0.73
*Turning in bed and adjusting bedclothes*			*0.47*	*0.42–0.53*
Gross motor function	0.89	0.88–0.91		
*Dressing and hygiene*			*0.38*	*0.33–0.43*
Turning in bed and adjusting bedclothes			0.57	0.51–0.62
Walking			0.93	0.91–0.96
Climbing stairs			0.98	0.96–1.00
Respiratory function	0.76	0.71–0.82		
Dyspnea			0.77	0.71–0.83
Orthopnea			0.99	0.93–1.07
Respiratory insufficiency			0.76	0.67–0.85
Bulbar function				
Fine motor function			0.23	0.16–0.31
Gross motor function			0.11	0.03–0.20
Respiratory function			0.43	0.35–0.52
Fine motor function				
Gross motor function			0.53	0.47–0.59
Respiratory function			0.35	0.26–0.43
Gross motor function				
Respiratory function			0.32	0.23–0.42

Estimates were obtained with the WLSMV estimator and standardized (STDYX); italic type indicates cross-loading items

### Reliabilities of the ALSFRS-R subscales

Reliabilities of ALSFRS-R subscales were estimated using CFA-based estimation in S2. Reliability coefficients with 95% confidence intervals are displayed in Table 4. Inspection of these coefficients shows that all subscales demonstrate acceptable to good internal consistency. The narrowness of these confidence intervals indicates that they can be regarded as providing accurate estimates of the internal consistency.

## Discussion

The primary objective of the present study was to assess the factorial validity of the ALSFRS-R. Our main finding is that the measurement model with a four-factor structure and two cross-loading items provides the best fit to the data. This is in contrast to previous studies that adopted a measurement model with a three-factor structure [5, 15, 16, 24], or a simple four-factor structure, i.e., without cross-loading items [17].

Cross-loading items have been listed in tables of previous reports [2, 3, 7, 18], but it seems their significance was not sufficiently recognized. These cross-loadings are, however, consistent with what clinicians come across in the assessment of ALS: that the items on dressing and hygiene and turning in bed and adjusting bed clothes measure activities that comprise both kinds of motor functioning. Including cross-loading items in the measurement model would, therefore, reflect the clinical reality. The application of our measurement model is, therefore, an adequate approach to assess disease severity in patients with ALS in existing data.

For the application of the ALSFRS-R in future studies, a revision of its item set is justified. Ideally, items that comprise more than one question are adapted or deleted from the item set during the development of measurement instruments. A revision of the ALSFRS-R item set could comprise items that are considered important indicators of disease severity by clinicians and patients. An example of a set of candidate items can be found in Wicks and colleagues [18], which was developed to measure disease severity in advanced stages of the disease.

Furthermore, our analyses indicate that measurement models of the ALSFRS-R with correlated factors describe the data significantly better than their equivalent with uncorrelated factors. The correlations between factors do, however, range from weak to modest, corroborating previous reports by Franchignoni and colleagues that the hypothesis that the ALSFRS-R is unidimensional is untenable [15, 16]. Due to this multidimensionality, ALSFRS-R items cannot validly be summed to obtain a total score that represents disease severity. Consequently, ALSFRS-R items constitute domain scores which reflect a profile of disease severity. Moreover, the application of these domain scores may allow a distinction between different trajectories of disease progression [24]. Our revision of the measurement model of the ALSFRS-R may, therefore, allow for a more adequate assessment of disease severity and disease progression in epidemiological studies and clinical trials.

With regard to reliability, our study supports the finding that all ALSFRS-R subscales demonstrated acceptable to good internal consistency.

Strengths of the present study are the use of both a calibration set and a validation set and the use of modification indices to investigate the measurement model of the ALSFRS-R. A weakness is that we only used data of patients administered a Dutch version of the ALSFRS-R. The generalizability of our findings should, therefore, be investigated in a cross-cultural study. Furthermore, the present study examined the measurement model in the ALS population. Given the heterogeneity of the disease [25], results might be different in subgroups of the population. Future studies should, therefore, assess measurement invariance of the ALSFRS-R between clinical subgroups of patients with ALS.

Our findings do complement earlier findings that ALSFRS-R items constitute a profile of domain scores, rather than a total score representing disease severity. Moreover, results of our study indicate that its measurement model should be revised to reflect the fact that the items on dressing and hygiene and turning in bed and adjusting bed clothes measure activities of daily living, which comprise both fine and gross motor functioning. Our findings may, therefore, allow for a more detailed analysis of disease severity and disease progression. Further studies on the measurement properties of the ALSFRS-R are necessary to expand the evidence on the appropriateness of its application.

## Electronic supplementary material

Below is the link to the electronic supplementary material.

**Online Resource 1.** M*plus* input for a confirmatory factor analysis of the ALSFRS-R using a measurement model with three-factor structure and correlated errors. **Online Resource 2.** M*plus* input for a confirmatory factor analysis of the ALSFRS-R using a measurement model with four-factor structure and two cross-loading items. **Online Resource 3.** Fit statistics for confirmatory factor analyses after multiple imputation (DOCX 27 kb)
