# Supplementary material for: Assessment of the factorial validity and reliability of the ALSFRS-R: a revision of its measurement model
Source: J Neurol. 2017 Jun 12;264(7):1413–20. doi: 10.1007/s00415-017-8538-4 (PMC5502060; doi:10.1007/s00415-017-8538-4)
Supplement: Supplementary file 1 — Online Resource 1. Mplus input for a confirmatory factor analysis of the ALSFRS-R using a measurement model with three-factor structure and correlated errors. Online Resource 2. Mplus input for a confirmatory factor analysis of the ALSFRS-R using a measurement model with four-factor structure and two cross-loading items. Online Resource 3. Fit statistics for confirmatory factor analyses after multiple imputation (DOCX 27 kb) [file 415_2017_8538_MOESM1_ESM.docx]

**SUPPLEMENTARY FILE**

**Article: The factorial validity of the ALSFRS-R: a revision of its measurement model**

By Leonhard A. Bakker, Carin D. Schröder, Michael A. van Es, Paul Westers, Johanna M. A. Visser-Meily, Leonard H. van den Berg.

**S1.** M*plus* input for a confirmatory factor analysis of the ALSFRS-R using a measurement model with three-factor structure and correlated errors.

|  |  |
| --- | --- |
| TITLE: | Confirmatory factor analysis (CFA) of the amyotrophic lateral sclerosis functional rating scale-revised (ALSFRS-R) using a measurement model with a three-factor structure and correlated errors i.e. between item 4 on handwriting and item 5 on handling food and utensils, and item 8 on walking and item 9 on climbing stairs. |
| DATA: | File = filename.csv; |
| VARIABLE: | Names =  ID  item1 item2 item3 item4 item5 item6  item7 item8 item9 item10 item11 item12;  Usevariables =  item1 - item12;  Categorical =  item1 - item12; |
| ANALYSIS: | Estimator = WLSMV; |
| MODEL: | ! specification of simple three-factor structure  f1 by item1* item2 item3;  f2 by item4* item5 item6 item7 item8 item9;  f3 by item10* item11 item12;  ! specification of correlated errors  item4 with item5;  item8 with item9;  ! fixing the variance at 1  f1@1 f2@1 f3@1;  ! specification of correlations between scales  f1 with f2-f3;  f2 with f3; |
| OUTPUT: | cinterval  modindices(all)  sampstat  standardized (STDYX)  residual; |
|  |  |

**S2.** M*plus* input for a confirmatory factor analysis of the ALSFRS-R using a measurement model with four-factor structure and two cross-loading items.

|  |  |
| --- | --- |
| TITLE: | Confirmatory factor analysis (CFA) of the amyotrophic lateral sclerosis functional rating scale-revised (ALSFRS-R) using a measurement model with a four-factor structure and two cross-loading items i.e. item 6 on dressing and hygiene and item 7 on turning in bed and adjusting bed clothes. |
| DATA: | File = filename.csv; |
| VARIABLE: | Names =  ID  item1 item2 item3 item4 item5 item6  item7 item8 item9 item10 item11 item12;  Usevariables =  item1 - item12;  Categorical =  item1 - item12; |
| ANALYSIS: | Estimator = WLSMV; |
| MODEL: | ! specification simple four-factor model  f1 by item1* item2 item3;  f2 by item4* item5 item6;  f3 by item7* item8 item9;  f4 by item10* item11 item12;  ! specification of cross-loading items  f2 by item7;  f3 by item6;  ! fixing the variance at 1  f1@1 f2@1 f3@1 f4@1;  ! specification of correlations between scales  f1 with f2-f4;  f2 with f3-f4;  f3 with f4; |
| OUTPUT: | cinterval  modindices(all)  sampstat  standardized (STDYX)  residual; |
|  |  |

**S3.** Fit statistics for confirmatory factor analyses after multiple imputation

Missing data was < 2,6% on all variables. To assess the potential effect of missing data on our analyses multiple imputation analyses were performed. The imputation process included all demographic and clinical variables and ALSFRS-R items. Fifty imputation datasets were generated with M*plus*. Comparison with Table 3 shows that these fit statistics are very similar to the fit statistics of the complete case analyses.

| Estimator | Model |  | *χ*^2^ | |  | *df* | |  | CFI | |  | TLI | |  | RMSEA | | BIC | |  |  |
| --- | --- | --- | --- | --- | --- | --- | --- | --- | --- | --- | --- | --- | --- | --- | --- | --- | --- | --- | --- | --- |
|  |  |  | S1 | S2 |  | S1 | S2 |  | S1 | S2 |  | S1 | S2 |  | S1 | S2 | S1 | S2 |  |  |
|  |  |  |  |  |  |  |  |  |  |  |  |  |  |  |  |  |  |  |  |  |
| MLMV | 1a |  | 882.82 | 806.93 |  | 54 | 54 |  | 0.76 | 0.78 |  | 0.70 | 0.73 |  | 0.14 | 0.13 | 24222 | 24387 |  |  |
|  | 1b |  | 458.06 | 411.64 |  | 48 | 48 |  | 0.88 | 0.89 |  | 0.84 | 0.85 |  | 0.11 | 0.10 | 23613 | 23862 |  |  |
|  | 1c |  | 261.06 | 225.43 |  | 47 | 47 |  | 0.94 | 0.95 |  | 0.91 | 0.93 |  | 0.08 | 0.07 | 23320 | 23595 |  |  |
|  | 1d |  | 158.93 | 132.32 |  | 46 | 46 |  | 0.97 | 0.98 |  | 0.95 | 0.96 |  | 0.06 | 0.05 | 23173 | 22464 |  |  |
|  |  |  |  |  |  |  |  |  |  |  |  |  |  |  |  |  |  |  |  |  |
|  | 2a |  | 758.34 | 714.13 |  | 54 | 54 |  | 0.79 | 0.81 |  | 0.75 | 0.77 |  | 0.13 | 0.13 | 24046 | 24254 |  |  |
|  | 2b |  | 635.67 | 612.27 |  | 51 | 51 |  | 0.83 | 0.84 |  | 0.78 | 0.79 |  | 0.12 | 0.12 | 23849 | 23120 |  |  |
|  | 2c |  | 343.57 | 320.01 |  | 50 | 50 |  | 0.91 | 0.92 |  | 0.89 | 0.90 |  | 0.09 | 0.08 | 23421 | 23707 |  |  |
|  | 2d |  | 238.57 | 231.91 |  | 49 | 49 |  | 0.95 | 0.95 |  | 0.93 | 0.93 |  | 0.07 | 0.07 | 23267 | 23583 |  |  |
|  |  |  |  |  |  |  |  |  |  |  |  |  |  |  |  |  |  |  |  |  |
| WLSMV | 1a |  | 6384.93 | 4700.63 |  | 54 | 54 |  | 0.74 | 0.76 |  | 0.68 | 0.71 |  | 0.39 | 0.33 |  |  |  |  |
|  | 1b |  | 746.56 | 641.47 |  | 48 | 48 |  | 0.97 | 0.97 |  | 0.96 | 0.96 |  | 0.14 | 0.13 |  |  |  |  |
|  | 1c |  | 441.79 | 571.49 |  | 47 | 47 |  | 0.98 | 0.99 |  | 0.98 | 0.98 |  | 0.10 | 0.10 |  |  |  |  |
|  | 1d |  | 232.86 | 196.44 |  | 46 | 46 |  | 0.99 | 0.99 |  | 0.99 | 0.99 |  | 0.07 | 0.07 |  |  |  |  |
|  |  |  |  |  |  |  |  |  |  |  |  |  |  |  |  |  |  |  |  |  |
|  | 2a |  | 1337.55 | 987.17 |  | 54 | 54 |  | 0.95 | 0.95 |  | 0.93 | 0.94 |  | 0.18 | 0.15 |  |  |  |  |
|  | 2b |  | 949.35 | 871.12 |  | 51 | 51 |  | 0.96 | 0.96 |  | 0.95 | 0.95 |  | 0.15 | 0.14 |  |  |  |  |
|  | 2c |  | 553.20 | 463.62 |  | 50 | 50 |  | 0.98 | 0.98 |  | 0.97 | 0.97 |  | 0.11 | 0.10 |  |  |  |  |
|  | 2d |  | 360.15 | 326.15 |  | 49 | 49 |  | 0.99 | 0.99 |  | 0.98 | 0.98 |  | 0.09 | 0.09 |  |  |  |  |
|  |  |  |  |  |  |  |  |  |  |  |  |  |  |  |  |  |  |  |  |  |

Note. Reported results are means over 50 datasets. All analyses had 50 successful computations for each statistic. All standard deviations were small, indicating that there was little difference between imputation datasets. BIC = Bayesian information criterion; CFI = comparative fit index; MLMV = maximum likelihood means and variance; RMSEA = root mean square error of approximation; S1 = calibration set; S2 = validation set; TLI = Tucker-Lewis fit index; WLSMV = weighted least squares means and variance.
